# Malignant ossifying fibromyxoid tumor of the tongue: case report and review of the literature

**DOI:** 10.1186/1746-160X-9-16

**Published:** 2013-06-24

**Authors:** Kouji Ohta, Masayuki Taki, Ikuko Ogawa, Shigehiro Ono, Kuniko Mizuta, Shinichi Fujimoto, Takashi Takata, Nobuyuki Kamata

**Affiliations:** 1 Department of Oral and Maxillofacial Surgery, Institute of Biomedical and Health Sciences, 1-2-3 Kasumi, Minami-Ku, Hiroshima 734-8553, Japan; 2 Center of Oral Clinical Examination, Hiroshima University Hospital, 1-2-3 Kasumi, Minami-ku, Hiroshima 734-8553, Japan; 3 Department of Oral and Maxillofacial Pathobiology, Institute of Biomedical and Health Sciences, 1-2-3 Kasumi, Minami-ku, Hiroshima 734-8553, Japan

**Keywords:** Ossifying fibromyxoid tumor, Tongue, Malignant, Recurrence

## Abstract

Ossifying fibromyxoid tumor (OFMT) is a rare mesenchymal neoplasm that arises in subcutaneous tissue, with that in the oral cavity extremely rare. We present a case of malignant OFMT in the tongue. A 26-year-old male noticed a painless mass in the tongue, which was extracted at a general hospital. Four years later, the tumor recurred and was resected at our department. Histologically, the recurrent tumor was composed of the closely packed cells positive for vimentin and S-100 proliferating in a nodular fashion. It showed high cellularity and mitotic activity. In the primary tumor, some tumor cells were arranged in a diffuse or cord-like manner within an abundant fibromyxoid matrix, along with a small amount of metaplastic ossification, corresponding with the histopathological characteristic of OFMT. Accordingly, a diagnosis of malignant OFMT arising in typical OFMT was established. This is the first reported case of malignant OFMT in the tongue. Long-term follow-up is needed for confirmation of prognosis and biological behavior.

## Introduction

Ossifying fibromyxoid tumor (OFMT) is an uncommon soft tissue neoplasm of uncertain origin, that is composed of relatively uniform round to ovoid cells often arranged in a corded or trabecular pattern and embedded in a fibromyxoid matrix [1-3]. In addition, a band of dense collagen with spicules of metaplastic bone is commonly encountered at the tumor periphery [1,2].

OFMT mostly arises in subcutaneous tissue or skeletal muscle of the extremities, while it has been reported at other sites, such as the trunk, head and neck, mediastinum and retroperitoneum at low frequency [1,2,4-6]. However, this tumor located in the oral cavity is extremely rare [2-4,7-9], with no cases of OFMT arising in the tongue previously reported.

OFMTs are presented as a slow growing painless, well-defined mass, and generally regarded as benign. Although most reported cases were cured by local excision, local recurrence rates ranging from 20% to 27% have been reported. In recent years, it was emphasized that a subset of OFMTs with conventional morphology displays atypical cytoarchitectural features, such as high cellularity or elevated mitotic activity and shows correspondingly more aggressive clinical behavior, which is called as atypical or malignant OFMTs [10,11]. We present a case of malignant OFMT arising in primary typical OFMT of the tongue.

## Case report

A 26-year-old male was referred to an otolaryngology at a general hospital in August 2003, because he became aware of a painless mass in the dorsal tongue before two weeks ago. The mass was a hard, smooth and 7 mm diameter, and was resected with narrow safety margin as diagnosis of a benign mesenchymal tumor. The histological diagnosis was chondromyxoid tumor, which is unusual, but that was not finally confirmed. After 4 years, he noticed a painless, mass gradually increasing in the same part, and consulted our oral and maxillofacial surgery clinic in January 2007.

**Figure 1:**
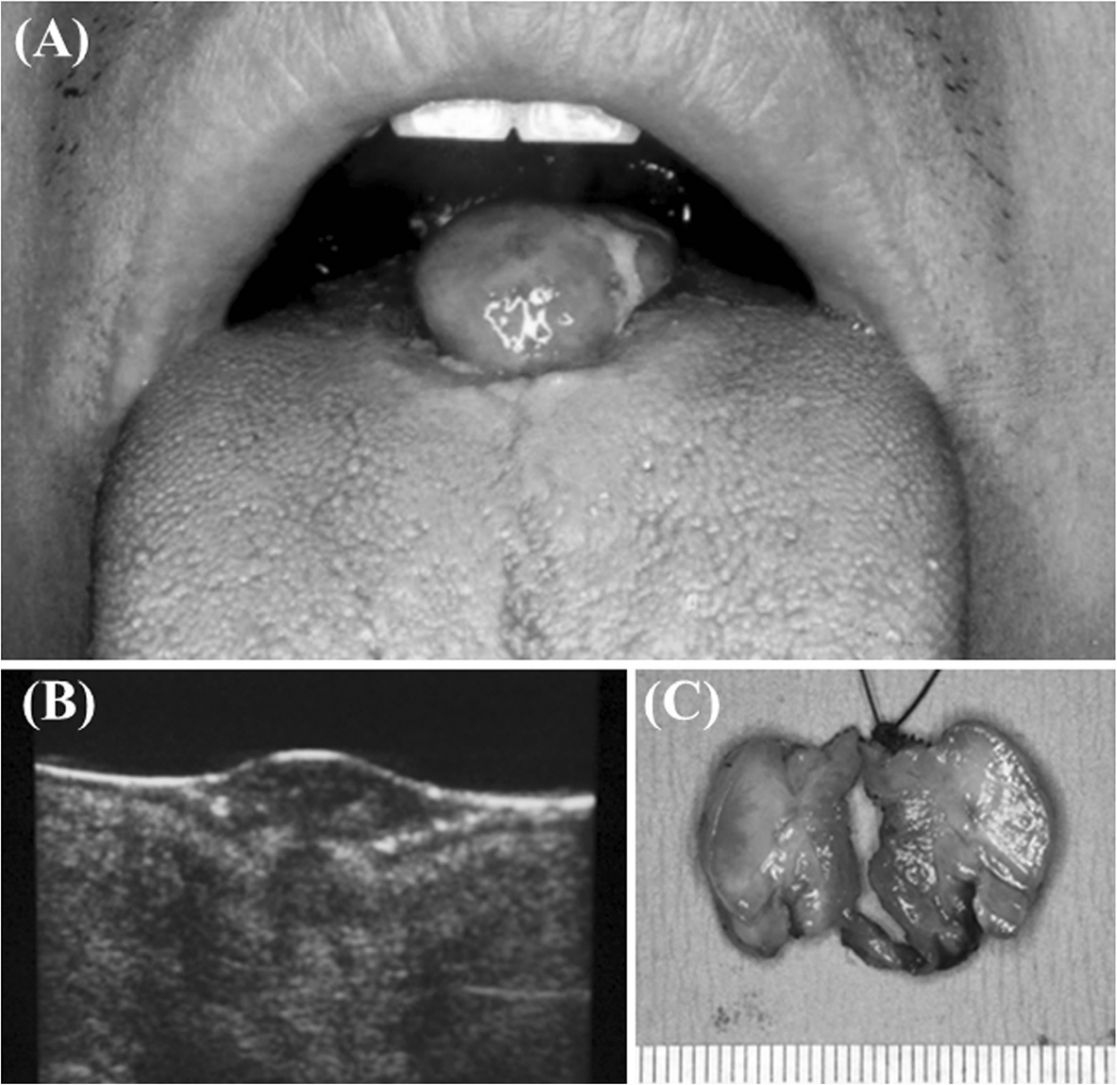
**Findings of the mass in the tongue. A**: Progressive swelling mass was founded at the tongue. **B**: Ultrasonographic findings showing a soft tissue mass. **C**: The cut section surface of the tumor was gray- white, solid, and firm in consistency.

The patient was healthy, with no family history of similar symptoms. The mobile pedunclated mass located at the center of the dorsum of tongue. The mass about 20 mm in diameter was firm in consistency, and had reddish and lobulated surface (Figure 1A). Ultrasonographic findings revealed a soft tissue mass in the tongue, which was 20 × 18 × 10 mm in size (Figure 1B). An incisional biopsy was performed based on the initial clinical diagnosis of soft tissue tumor of the tongue, and histological findings suggested a benign mesenchymal tumor as a possible diagnosis.

**Figure 2:**
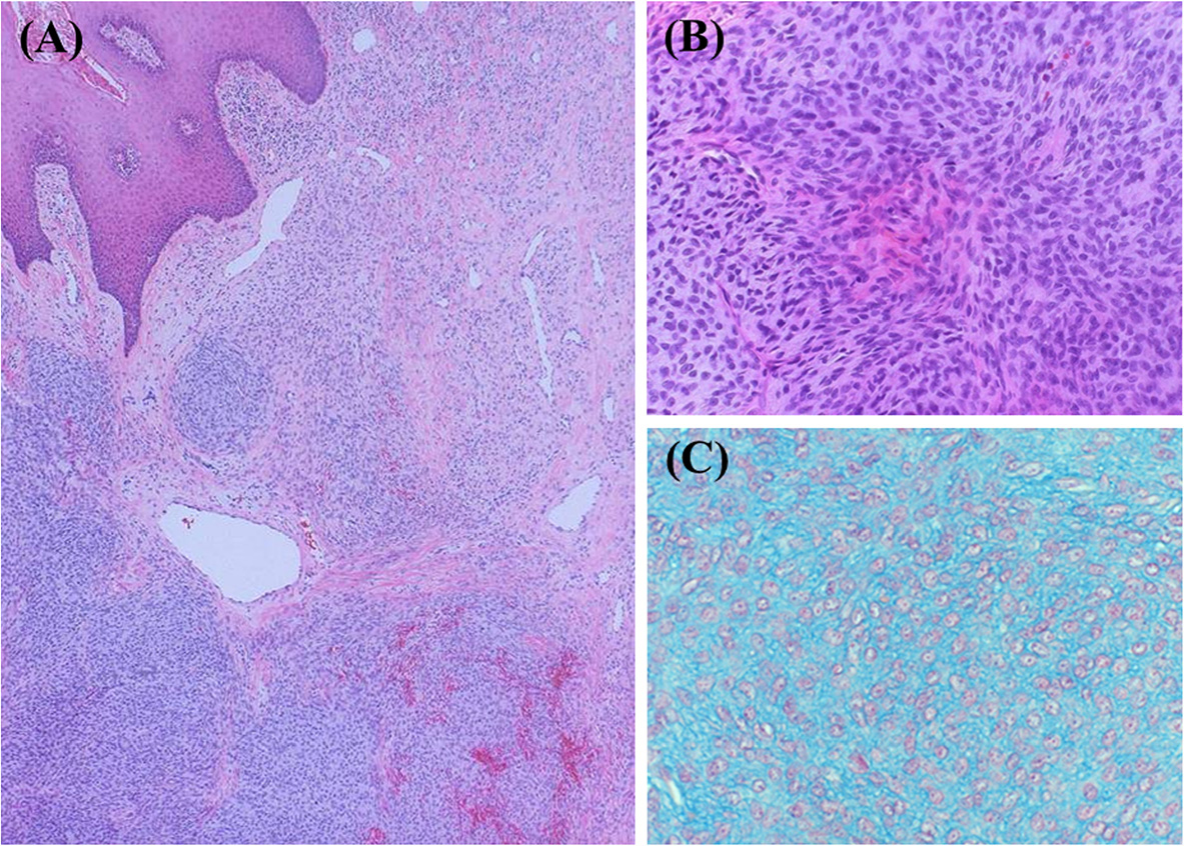
**Histopathological features of the recurrent tumor. A**: Multinodular growth pattern without encapsulationis shown. (20x, Hematoxilin-Eosin staining) **B**: The tumor was composed of solid proliferating uniform short spindle or ovoid cells with vesicular nuclei, and a small amount of cytoplasm (100x, Hematoxilin-Eosin staining). **C**: The tumors cells were separated by alcian blue positive mucoid material in small areas. (200x, Alcian blue staining).

Under general anesthesia, the tumor was extirpated with a safety margin of about 3 mm. Macroscopically, the tumor was solid and lobular with a grayish white cut surface, and measured 20 mm at the greatest diameter (Figure 1C). Histologically, the tumor was located in subepithelial connective tissue and proliferated in a multinodular fashion (Figure 2A). The mass was relatively well circumscribed but not encapsulated. It was composed of solid and lobular proliferation of uniform short spindle or ovoid cells with vesicular nuclei and indistinct small amounts of eosinophilic or vacuolated cytoplasm (Figure 2B). In most areas, the tumor cells were closely packed and showed high cellularity. In some small areas, they were arranged in a diffuse haphazard or sheet-like fashion with an alcian-blue positive myxoid or collagenous stroma (Figure 2C).

**Figure 3:**
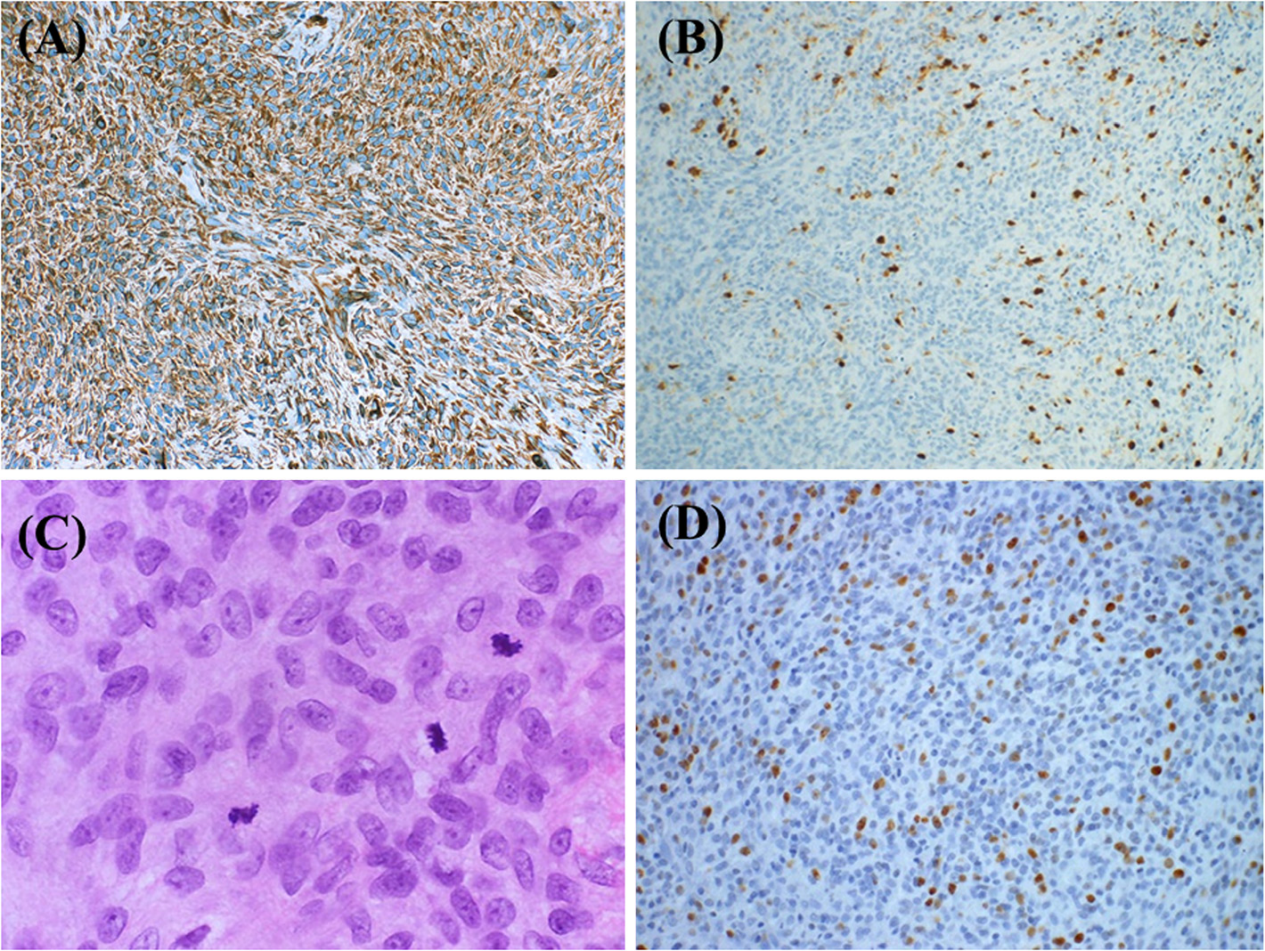
**Immunohistochemical characteristics and proliferative activity of the recurrent tumor. A**: Most tumor cells displayed immunoreactivity for vimentin (40x, Immunohistochemical staining). **B**: S-100 protein was also expressed in some tumor cells (40x, Immunohistochemical staining). **C**: Mitotic figures were easily found (400x, Hematoxilin-Eosin staining). **D**: Ki-67 positive cells were frequently observed (100x, Immunohistochemical staining).

A variety of lesions with fibromyxoid matrix was considered as differential diagnosis including myoepithelioma/myoepithelial carcinoma, ectomesenchymal chondromyxoid tumor of the anterior tongue, epithelioid nerve sheath tumors and epithelioid smooth muscle tumor. For the establishment of the diagnosis, immunohistochemical analysis was performed using various antibodies (Table 1). The tumor cells expressed vimentin (Figure 3A) and some of them were also positive for S-100 protein (Figure 3B). Other markers were not expressed. In spite of scant cellular and nuclear polymorphism, the mitotic activity was relatively high, i.e., 2 mitotic figures per 10 high-power fields (HPFs) with areas showing more than 3 mitoses in 1 HPF (Figure 3C), and the Ki-67 labeling index was about 7% (Figure 3D). Detailed analyses were performed, but it was still difficult to further classify this tumor due to the unusual morphological and immunohistochemical features.

**Table 1:** Primary antibodies used in the analysis of OFMT

**Antigen**	**Antibody clone(s)**	**Source of antibody**
Vimentin	V9	Dako Cytomation, Carpinteria, CA
S100 protein	polyclonal	Dako Cytomation
GFAP	polyclonal	Dako Cytomation
Cytokeratins	AE1/AE3	Invitrogen, Carlsbad, CA
Cytokeratins	CAM5.2	Becton Dickinson, Frankin Lakes, NJ
α-SMA	1A4	Dako Cytomation
Calponin	CALP	Dako Cytomation
Desmin	D33	Dako Cytomation
CD68	KP1	Dako Cytomation
CD34	NU-4A1	Nichirei Biosciences Inc, Tokyo, Japan
P63	4A4	Dako Cytomation
Ki-67	MIB-1	Dako Cytomation

**Figure 4:**
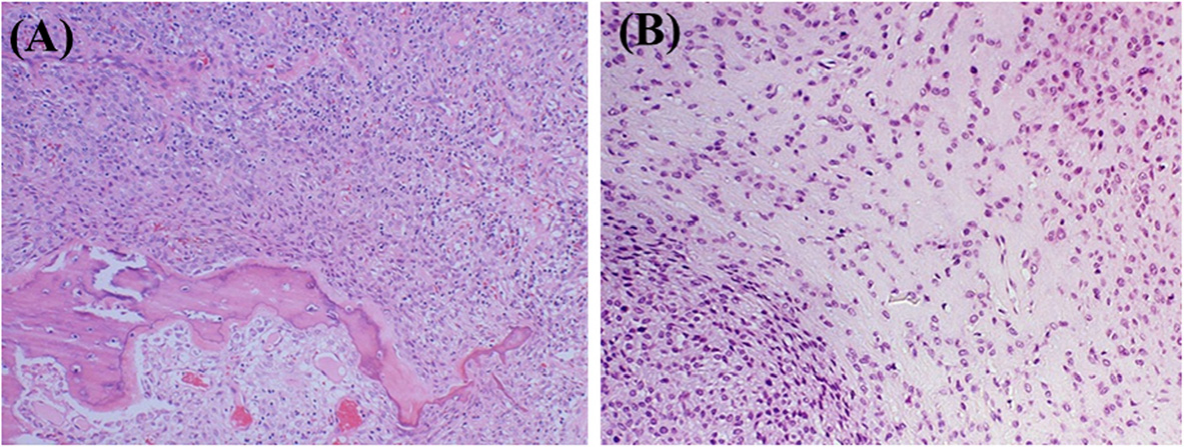
**Histopathological features of the primary tumor. A:** Small amounts of metaplastic ossification and osteoid formation were found in the primary tumor (40x, Hematoxilin-Eosin staining). **B:** Cords of tumor cells were seen suspended within a fibromyxoid matrix (100x, Hematoxilin -Eosin staining).

We made a tentative diagnosis of mesenchymal tumor with low-grade malignancy. Since a final diagnosis could not be established, histological specimens of the primary tumor were obtained from the pathological department of the hospital where the patient underwent the initial treatment. Although the histological appearance was essentially identical to that of the present extracted tumor, small amounts of metaplastic ossification and osteoid was found in the primary tumor (Figure 4A). In addition, the tumor cells were arranged in a diffuse or cord-like manner with abundant fibromyxoid matrices (Figure 4B) in some areas. These features and immunohistochemical natures are consistent with those of typical OFMT. The recurrent tumor, however, showed higher cellularity without intervening matrix and high mitotic activity of more than 2 mitotic figures/10HPFs, which deviate form the histopathologic features of typical OFMT and are histologic parameters suggestive for increased risk of local recurrence and metastasis. As a result, the final diagnosis of malignant OFMT arising in typical OFMT was established, although high nuclear grade (defined as irregular nuclear contours, coarse chromatin, and macronucleoli) was not observed.

## Discussion

OFMT is a rare mesenchymal neoplasm initially reported by Enzinger *et al*. in 1989, in which, approximately 70% of the examined cases showed the subcutaneous tissue involvement in the lower or upper limbs, whereas OFMT in the head and neck region only accounted for about 13% of those cases [1]. Involvement in the oral cavity is extremely rare, with only 7 cases have reported in the soft palate, buccal mucosa, vestibule, lip, and gingiva (Table 2) [3,4,7-9]. There are no known previous cases of this tumor arising in the tongue or malignant OFMT in the oral cavity.

**Table 2:** Reported cases of ossifying fibromyxoid tumor in the oral cavity

**Case**	**Age**	**Gender**	**Location**	**Size (cm)**	**Clinical resentation**	**Mitosis**	**Atypia**	**Osteoid formation**	**Recurrence**	**Follow-up**	**Reference**
1	N / D	N / D	Lower lip	N / D	N / D	N / D	N / D	N / D	N /D	N / D	[2]
2	14	F	Between buccal and gingival mucosa	3.5	Nodular swelling	Less than 2/10 HPFs	-	+	N /D	N / D	[3]
3	67	F	Mandible vestibule	1	N / D	N / D	N / D	N / D	No	3 yrs	[4]
4	37	M	Soft palate	4.5	N / D	N / D	N / D	N / D	No	1.5 yrs	[4]
5	41	M	Inner cheek	1.5	N / D	0-1 / 10 HPFs	-	N / D	No	N / D	[7]
6	39	M	Lip	1.5	N / D	0-11 / 10 HPFs	-	N / D	No	N / D	[7]
7	21	F	Mandibular gingiva	6	Reddish, lobulated surface	No mitosis	-	+	No	7 mos	[8]
8	26	M	Toungue	2	Painless, firm, reddish, lobulated surface	More than 2/10 HPFs	-	+	yes	4 yrs	Present case

M = male, F = female, N/D = detail not provided, yr(s) = years, mo(s) = month(s), HPFs = high power fields.

Clinically, OFMT is usually presented as a slow growing painless mass. It is most common in the fifth to seventh decades of life, with male predominance [1,2]. The masses are usually less than 10 cm in diameter [1,2]. However, Harris et al. reported 9 cases of giant OFMTs, which had diameters greater than 10 cm [12]. Most of OFMT occurring in oral cavity range in the size from 60 mm to 10 mm (Table 2), which was also seen in the present case [2].

Microscopically, OFMTs are composed of uniform round, ovoid or spindle-shaped cells arranged in nests and cords with fibromyxoid stroma partially surrounded by shell of mature bone. Immunohistochemical findings have revealed that 74% of these tumor are positive for vimentin and 94% positive for S-100 protein [1,2], features consistent with neural crest lineage or cartilaginous differentiation. The primary tumor in the present case showed the characteristics of OFMT. The mitotic activity and nuclear grade were low, although the areas exhibiting hypercellularity were mixed. The recurrent tumor maintained the morphological features of OFMT, but was composed of closely packed cells with higher cellularity and mitotic activity in spite of inconspicuous cell atypia and lacked bone or osteoid formation. Clinicopathologic study of 70 cases of OFMT by Folpe indicated that the tumors having 1) high nuclear grade or 2) high cellularity and mitotic activity >2 mitotic figures/50HPF have a substantial risk of metastasis [10]. The histological features of the recurrent tumor are consistent with those of malignant OFMT. After revealing the features of the primary tumor, the recurrent tumor was finally diagnosed as malignant OFMT arising in typical OFMT. In 15 cases of malignant OFMTs reported by Graham et al., malignant foci could be identified in subsequent local recurrences of initially typical OFMTs [13]. The percentage of the cases with bone formation in malignant OFMT is low (47%) than that in typical one (60%).

Awareness of OFMT occurring in the oral cavity is important to avoid confusion with other tumors with a fibromyxoid matrix. Differential diagnosis of OFMT in the oral cavity includes myoepithelioma and ectomesenchymal chondromyxoid tumor (ECT) of the anterior tongue. The absence of epithelial and myoepithelial markers excludes myoepithelioma as a diagnostic consideration. ECT is an extremely rare benign tumor that usually arises in the submucosa of the anterior dorsum of the tongue. Its histogenesis is unclear, though it is possibly derived from undifferentiated ectomesenchymal progenitor cells that have migrated form the neural crest. ECT is composed of round, fusiform or polygonal cells with a chondromyxoid matrix, resembling the features of OFMT. However, the tumor cells of ECT strongly express GFAP, which is different from the immuno-profile of OFMT. Furthermore, ECT lacks bone and osteoid formation. The line of differentiation of OFMT remains controversial. Graham et al. described that expression of neuron-related markers, in addition to Schwann cell and cartilage-associated markers, suggests a “scrambled” phenotype in this tumor.

Genetic studies on OFMT are limited. Graham et al. suggested that OFMT develops primarily through inactivation of the *SMARCB1* (also known as *INI*-*1* or *SNF5*) gene in chromosome band 22q11 [13]. Furthermore, gene expression profiling showed typical and malignant OFMTs to cluster together. Whereas, Gebre-Medhin et al. reported that the pathogenetic basis of OFMT frequently involves rearrangement of the PHF1 gene, suggesting that epigenetic deregulation of PRC2 target genes is crucial for tumor development regardless of types such typical, atypical and malignant [14]. Therefore, cytogenetic assay and FISH tests for reaarangment of these genes may be useful diagonstic tools in ambiguaous cases of OFMT.

In the series of Graham et al., all patients with typical and atypical OFMT were alive without local recurrence and metastasis, but adverse events were seen in 33% of patients with malignant OFMT, including 2 patients with local recurrences, 3 patients with distant metastases, and 3 deaths from disease [13]. Therefore, careful follow-up examinations will be mandatory for the present patients.

## Conclusion

We present the first reported case of malignant OFMT with recurrence arising in typical OFMT of the tongue. In the primary tumor, some tumor cells were arranged in a diffuse or cord-like manner within an abundant fibromyxoid matrix, along with a small amount of metaplastic ossification, corresponding with the histopathological characteristic of OFMT, whereas the recurrent tumor was regarded as a malignant OFMT because of increased cellularity and mitotic activity. Therefore, long-term follow-up is needed for confirmation of the biological behavior.

## Consent

The authors declare that the patient had given consent for the case report to be published.

## Competing interest

The authors declare that they have no competing interests.

## Authors’ contribution

KO and MT performed surgery and drafted the manuscript. IO performed histopathological examination and immunohistochemical staining and drafted the manuscript. SO, KM and FS treated the patient and helped to draft the manuscript. TT and NK conducted a review of literature and helped to draft the manuscript. All authors read and approved the final manuscript.
